# Effect of Chocolate and Yerba Mate Phenolic Compounds on Inflammatory and Oxidative Biomarkers in HIV/AIDS Individuals

**DOI:** 10.3390/nu8050132

**Published:** 2016-05-23

**Authors:** Aline A. Petrilli, Suelen J. Souza, Andrea M. Teixeira, Patricia M. Pontilho, José M. P. Souza, Liania A. Luzia, Patricia H. C. Rondó

**Affiliations:** 1 Nutrition Department, School of Public Health, University of São Paulo, São Paulo, CEP 01246-904, Brazil; alinepetrilli@gmail.com (A.A.P.); suelenjorge@usp.br (S.J.S.); andreamariana12@gmail.com (A.M.T.); patty.dmp@gmail.com (P.M.P.); lianialuzia@usp.br (L.A.L.); 2 Epidemiology Department, School of Public Health, University of São Paulo, São Paulo, CEP 01246-904, Brazil; jmpsouza@usp.br

**Keywords:** polyphenols, chocolate, yerba mate, oxidative stress, inflammation, HIV/AIDS

## Abstract

Flavonoids in cocoa and yerba mate have a beneficial role on inflammation and oxidative disorders. Their effect on HIV individuals has not been studied yet, despite the high cardiovascular risk of this population. This study investigated the role of cocoa and yerba mate consumption on oxidative and inflammatory biomarkers in HIV+ individuals. A cross-over, placebo-controlled, double-blind, randomized clinical trial was conducted in 92 individuals on antiretroviral therapy for at least six months and at viral suppression. Participants were randomized to receive either 65 g of chocolate or chocolate-placebo or 3 g of yerba mate or mate-placebo for 15 days each, alternating by a washout period of 15 days. At baseline, and at the end of each intervention regimen, data regarding anthropometry, inflammatory, oxidative and immunological parameters were collected. High-sensitivity C-reactive protein, fibrinogen, lipid profile, white blood cell profile and thiobarbituric acid reactive substances were assessed. There was a difference between mean concentrations of HDL-c (ANOVA; *p* ≤ 0.05) among the different regimens: dark chocolate, chocolate-placebo, yerba mate and mate-placebo. When a paired Student *t*-test was used for comparisons between mean HDL-c at baseline and after each regimen, the mean concentration of HDL-c was higher after supplementation with dark chocolate (*p* = 0.008).

## 1. Introduction

Cardiovascular complications are among the most common comorbidities in individuals with HIV/AIDS and are associated with a higher risk of mortality [1,2]. Different protein components of HIV and the chronic use of antiretroviral therapy (ART), in conjunction with metabolic alterations (e.g., lipid profile changes), cause an increase in oxidative stress and inflammatory markers, and these factors are likely to predispose to chronic diseases, such as cardiovascular disorders [3,4].

Reduced high-density lipoprotein (HDL-c) and increased low-density lipoprotein (LDL-c) levels are characteristics of individuals with HIV/AIDS. HDL-c exerts anti-inflammatory activity, and LDL-c is characterized by a high oxidative capacity [5]. The increase in reactive oxygen species (ROS) induces the oxidation of LDL-c particles, predisposing to the formation of electronegative subfractions(LDL(−))that possess cytotoxic and proinflammatory properties. These factors lead to the exacerbation of oxidative stress related to an increased inflammatory response and progression of the disease [5,6].

The reversal of the symptoms of oxidative stress depends on the redox balance of the organism. However, changes in CD4 T lymphocytes reduce the antioxidant capacity of individuals with HIV/AIDS [7]. Furthermore, viral proteins gp120 and Tat negatively influence the concentrations of the antioxidant enzymes glutathione reductase and glutathione peroxidase [2]. Another factor that seems to influence oxidative stress and inflammation in individuals with HIV/AIDS is ART. Known side effects of the chronic use of ART are accumulation and redistribution of body fat. This increase in adiposity is related to an increase in inflammatory markers, such as high-sensitivity C-reactive protein (hs-CRP) and fibrinogen [8,9,10].

Some foods, such as cocoa and yerba mate, are rich in flavonoids, compounds that reduce oxidative and inflammatory markers, such as thiobarbituric acid reactive substances (TBARS) and hs-CRP [11,12,13]. The main phenolic compounds found in cocoa are within the classes of flavonoids and tannins. The flavonoids include flavonols, anthocyanins, flavones and flavanones [14,15]. The tannins are a complex series of procyanidins formed from the condensation of monomeric units of catechins and epicatechins [16]. In yerba mate, the main phenolic compounds identified are the caffeoyl derivatives and flavonoids that include quercetin, kaempferol and rutin [17].

*In vitro* and *in vivo* studies have shown that the flavonoids found in these foods increase the antioxidant capacity of the organism, inhibit the oxidation of LDL-c and reduce the blood concentrations of this particle, with a concomitant increase in HDL-c [18,19,20,21].

To our knowledge, there are no studies in the international literature evaluating the impact of cocoa and yerba mate consumption on the oxidative and inflammatory profile of HIV-positive individuals.

## 2. Experimental Section

### 2.1. Ethical Statement

The present study was registered in the Netherlands National Trial Register (NTR) under Number 3176. The project was approved by the Ethics Committee of the School of Public Health (no. 2018/2009), Faculty of Medicine, University of São Paulo (no. 0753/2010), and of the Emilio Ribas Institute of Infectology (no. 44/2012), São Paulo, Brazil. The procedures were in accordance with the Declaration of Helsinki.

### 2.2. Experimental Design and Subjects

A randomized, double-blind, placebo-controlled cross-over clinical trial was conducted from 2011 to 2015. The study included 92 individuals infected with HIV of both genders aged 19 to 59 years, who had an undetectable viral load (<500 copies/mL) and who had used ART for at least 6 months. Criteria for exclusion were a history of cardiovascular diseases, cerebrovascular accident, kidney disease, Chagas’ disease, severe intestinal disease, hypothyroidism, hepatic insufficiency, active hepatitis B or C, malignant neoplasms, opportunistic infection, pregnancy, use of illicit drugs, a diagnosis of diabetes or hypertension, vegetarianism, active participation in another nutritional intervention study, supplement intolerance and use of anti-inflammatory drugs.

### 2.3. Data Collection

**Figure 1:**
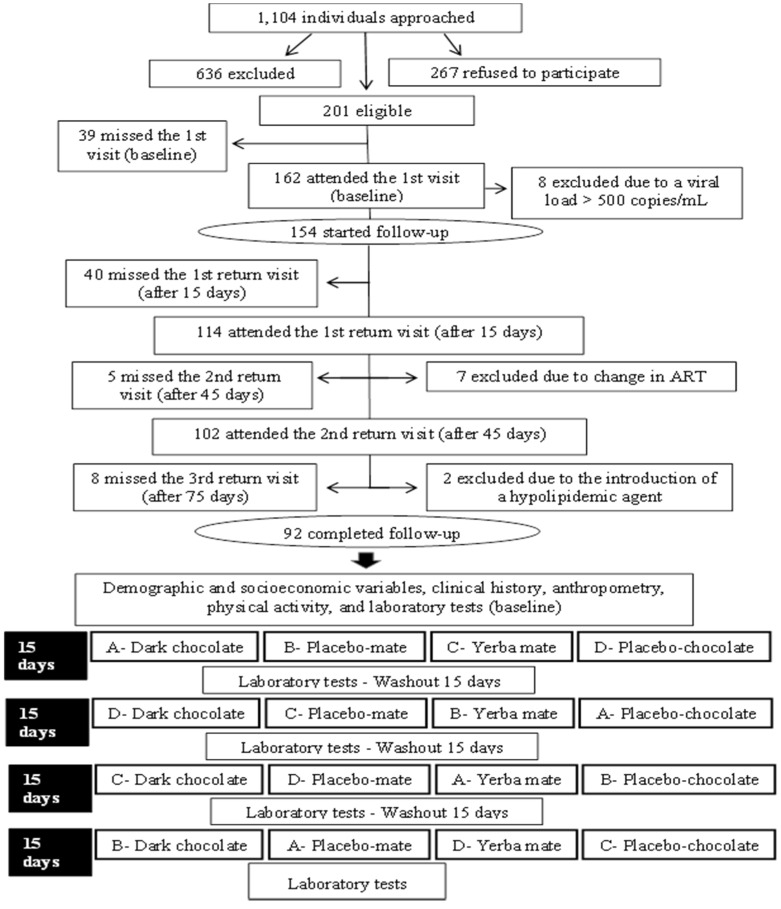
Flow diagram of the clinical trial.

The data collected were stored in the EpiData software (EpiData Association, Odense, Denmark) and transferred to the Microsoft Excel^®^ program (Microsoft, NM, USA, 2010). Figure 1 illustrates the flow diagram of the study.

As can be seen in Figure 1, the participants were evaluated in five phases during the study: (1) 1st visit (baseline); (2) 1st return (after 15 days); (3) 2nd return (after 45 days); (4) 3rd return (after 75 days); (5) 4th return (after 105 days).

During the 1st visit, demographic and socioeconomic variables, adherence to ART [22] and the clinical history of the participants were obtained, checking them with patients’ records. Anthropometric and biochemical data were collected during the first visit and during the return visits.

### 2.4. Supplementation

Chocolate bars were developed at the Institute of Food Technology (Campinas, Brazil) and produced by the researchers in an experimental unit of “JAF INOX^®^ Integrated Systems Cocoa to Chocolate” (São Roque, Brazil). The liquor of cocoa and cocoa butter used for the manufacture of chocolate bars were acquired at “Barry Callebault Brazil Industry and Trade in Food Products” (Zürich, Switzerland). Mate tea and its placebo were developed at the Bromatology Laboratory of the School of Public Health, University of São Paulo, Brazil. Mate tea was formulated using yerba mate soluble granulate from “Leão Alimentos e Bebidas^®^” (São Paulo, Brazil), added maltodextrin (New Millen^®^, São Paulo, Brazil) and peach artificial flavor (“Duas Rodas”^®^, Jaragua do Sul, Brazil) according to the methodology described by Mazzafera [23] and Bastos *et al.* [17]. For the placebo, a mixture of maltodextrin, caramel food coloring (Corn Products^®^, Conchal, Brazil) and peach artificial flavor (“Duas Rodas^®^”) was used. The caramel colorant used for the placebo tea was identical to the natural color of yerba mate.

The participants received 65 g of a chocolate bar containing 36 g cocoa, corresponding to an average of 2864 mg polyphenols (approx. 550 mg/day of flavonoids) [24] or 65 g white chocolate as placebo. Both types have a similar nutritional composition and only differ in the presence or absence of phenolic compounds. In the case of yerba mate, 3 g of a preparation containing soluble yerba mate plus maltodextrin and artificial peach flavor, corresponding to 107 mg/g total phenols, was offered. The placebo-mate was formulated with 3 g of a solid preparation containing maltodextrin, caramel coloring and artificial peach flavor. Potassium sorbate (0.003 g) was added to both formulations for conservation. All supplements were packed in metallized Biaxially Oriented Polypropylene(BOPP) bags.

The intervention period lasted 60 days divided into four phases of 15 days each, corresponding to four different supplementation regimens. After each regimen, there was a washout period of 15 days before the beginning of the next regimen, for a total of approximately 105 days of follow-up. The supplementation regimens were randomized at baseline, ensuring that each participant received one of the four sequences of supplements: Sequence A = 1,4,3,2; Sequence B = 2,3,4,1; Sequence C = 3,2,1,4; and Sequence D = 4,1,2,3; where 1 = dark chocolate, 2 = placebo-mate, 3 = yerba mate, 4 = placebo-chocolate (Figure 1).

### 2.5. Anthropometry

The following variables were obtained: weight (kg) and height (cm) for the determination of body mass index (BMI), waist circumference (WC) and body composition data (fat mass percentage). Weight and body composition were determined with a Tanita^®^ MC-180 MA tetrapolar segmental composition analyzer (Tokyo, Japan), with a capacity of 270 kg. Height was measured with a Seca^®^ stadiometer (80 to 220 cm; Hamburg, Germany) to the nearest 0.1 mm.

### 2.6. Laboratory Tests

During the first visit and the return visits, 20 mL blood were collected by peripheral venipuncture into dry tubes and tubes containing 1.0 mg/mL EDTA (BD, Brazil). Serum and plasma were separated by centrifugation at 300 rpm for 15 min at room temperature. The following parameters were used to evaluate the inflammatory and oxidative profile: hs-CRP, fibrinogen, TBARS, lipid profile parameters and white blood cell count.

hs-CRP: particle-enhanced immunonephelometry using CardioPhase^®^hs-CRP reagent (Siemens, Malvern, PA, USA). The hs-CRP values were classified according to Pearson *et al.* [25].

Fibrinogen: Clauss method (clotting activity) using the Multifibren U kit (Dade Behring^®^, Deerfield, IL, USA). The fibrinogen cutoff values recommended by Handin were used [26].

TBARS: one milliliter of a solution containing thiobarbituric acid (0.046 M), trichloroacetic acid (0.92 M) and hydrochloric acid (0.25 M) was added to 50 μL plasma and incubated in a water bath (100 °C) for 30 min. The samples were then centrifuged at 8000× *g* for 15 min at 4 °C (Sigma^®^ 318-K).Absorbance (535 nm) of the supernatant was read in a Shimadzu UV 1650 PC spectrophotometer. For quantification, a standard curve was constructed using 1,1,3,3-tetraethoxypropane (TEP) (96% purity) over a concentration range of 0.2 to 4 μmol/L [27,28,29]. Values are expressed as μmol TBARS/L plasma. No cutoff values are available for TBARS. The increase or decrease in the oxidative process is determined based on the baseline values of this marker.

Lipid profile: cholesterol oxidase-phenol aminoantipyrine and glycerol-3-phosphate oxidase- phenol aminoantipyrine (CHO/PAP and GPO/PAP) enzymatic-colorimetric methods using Roche^®^ kits (Basel, Switzerland). LDL-c values were calculated using the equation of Friedewald *et al.* [30]: LDL-c = total cholesterol − (VLDL-c + HDL-c), where VLDL-c = triglycerides/5. The values proposed by the National Cholesterol Education Program–Adult Treatment Panel III [31] were defined as normal values.

White blood cell count: flow cytometry by dispersion and fluorescence inXT-2000i equipment (Sysmex Corporation, Kobe, Japan) [32].

Immunological and virological analysis: Qualitative and quantitative analysis of T lymphocytes (CD4 and CD8) was performed in a BD FACSCalibur^®^ flow cytometer (Wembley, England) after these cells were labeled with specific fluorescent monoclonal antibodies. The viral load was determined by the Branched DNA 3.0 method (Versant^®^, Freemont, CA, USA). This assay can be used to directly quantify HIV-1 RNA in a 340 dDNA Analyzer (Bayer^®^, Leverkusen, Germany). Viral and lymphocyte counts were classified according to the parameters of Mellors *et al.* [33].

### 2.7. Statistical Analysis

First, descriptive analysis of the pattern of distribution of each variable was performed. Qualitative variables are reported as frequencies and quantitative variables as means and standard deviation.

Analysis of variance (ANOVA) was used for cross-over analysis to evaluate differences in intraindividual responses between interventions (dark chocolate/yerba mate and placebos) in relation to the outcome variables. The pkcross procedure of the Stata 11.0 software (Stata Corporation, College Station, TX, USA) was utilized for this analysis, assuming a washout period of 15 days, to avoid a cumulative effect (carryover) between interventions. The Bonferroni modified *t*-test was utilized for comparisons of the inflammatory and oxidative markers between the four different interventions. The paired Student *t*-test was used for comparisons between the inflammatory and oxidative markers at baseline and after receiving the different interventions. A *p*-value ≤ 0.05 was considered significant.

## 3. Results

Of the 1104 subjects initially approached, 636 did not meet the eligibility criteria and 267 refused to participate. Thus, 201 subjects started the study, and 92 completed all supplementation regimens (Figure 1).

As can be seen in Table 1, age was the only characteristic that differed significantly between subjects who completed the study and those who did not. Age is a factor that influences the inflammatory response of the organism [34]. However, a comparison of mean hs-CRP, fibrinogen and HDL-c between the two groups permits us to conclude that the inflammatory profile was similar, *i.e.*, the age difference was not sufficient to generate differences in the inflammatory markers and parameters analyzed.

Most of the 92 participants who completed the study were males (63%), with a mean age of 45 years. Approximately half the subjects (46.7%) reported being white, 43.5% were single and 51.1% were from the State of São Paulo. Regarding education level, 57.6% had completed high school, and 32.6% had a higher education. More than half the participants (53.3%) did not have children, and 67.4% were employed at the time of the study. The mean per capita income was R$1505.2 (approximately US$465.57).

The majority of the anthropometric parameters were within the normal range. Mean weight, height and BMI were 66.8 (±12.5) kg, 168.8 (±8.9) cm and 23.4 (±3.9) kg/m^2^, respectively. Regarding the classification of WC according to gender, although the WC of most participants (62%) was within the normal range, 61.8% of women *versus* 24.1% of men had abdominal obesity. No significant changes in the anthropometric characteristics were observed during the study.

Analysis of smoking habits showed that most participants did not use tobacco (66.3%), and 16.3% were ex-smokers. Habitual consumption of alcoholic beverages (at least once a month) was reported by 50% of the participants. Most subjects (90.2%) did not use illicit drugs.

The mean time of HIV infection was 13.3 (±5.1) years, with the onset of therapy after 10.7 (±5.2) years, and a mean duration of current therapy of 3.6 (±3.3) years. Regarding treatment compliance, 95.7% of the subjects reported the ingestion of all antiretroviral drugs according to medical prescription on the three days preceding the date of the interview.

Analysis of inflammatory and oxidative variables showed that 66.3% of the participants had hs-CRP values above the recommended value; of these, 22.8% had an increased risk of developing cardiovascular diseases [28]. Fibrinogen levels were within the normal range in 71.7% of the subjects.

Most participants had adequate HDL-c levels (62.1% of men and 61.8% of women). Leukocyte, neutrophil, lymphocyte and monocyte counts were within the normal range in most subjects (87%, 92.4%, 97.8% and 95.7%, respectively).

**Figure 2:**
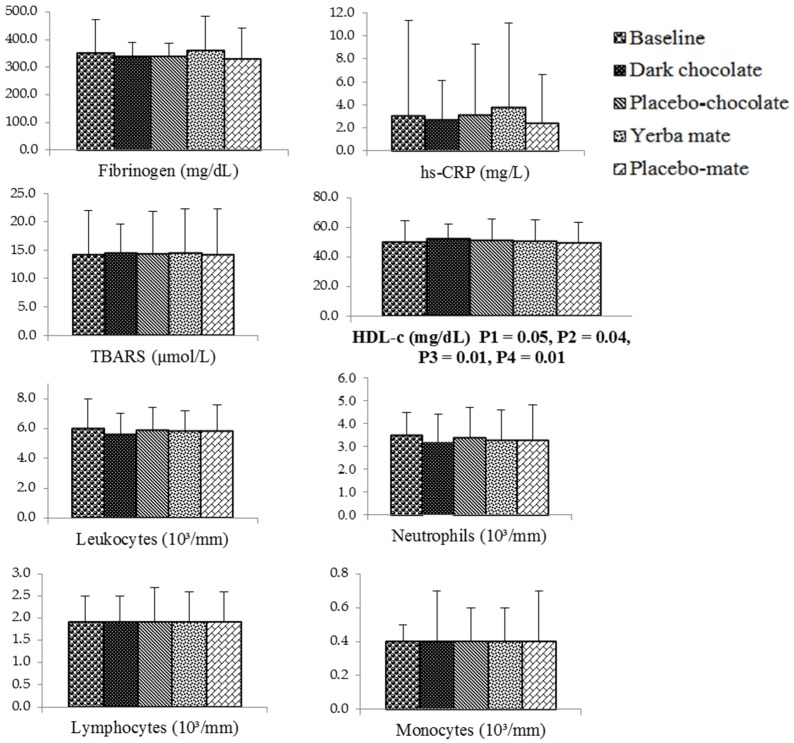
Bar graphs of inflammatory and oxidative variables of individuals with HIV/AIDS undergoing antiretroviral therapy at baseline and after receiving the different supplements (n* = 92) ANOVA: P1, comparison of means between the four supplements. Bonferroni modified *t*-test: P2, dark chocolate *versus* yerba mate; P3, dark chocolate *versus* placebo-mate. Paired Student *t*-test: P4, baseline *versus* dark chocolate. hs-CRP, high-sensitivity C-reactive protein; TBARS, thiobarbituric acid reactive substances; HDL-c, high-density lipoprotein-cholesterol. n*: Fibrinogen (placebo-chocolate, *n* = 90, yerba mate, *n* = 89, placebo-mate, *n* = 89); neutrophils (yerba mate, *n* = 91) and monocytes (yerba mate, *n* = 91).

Figure 2 shows the comparison between the four different supplementation regimens. A significant difference was observed for HDL-c (ANOVA, *p* = 0.047). This analysis has considered simultaneously all four regimens of the study (dark chocolate, placebo chocolate, yerba mate and placebo mate). Mean values of HDL-c (mg/dL) at baseline and after receiving dark chocolate, placebo chocolate, yerba mate and placebo mate were, respectively: 49.8 (14.8), 52.1 (15), 50.6 (15), 50.3 (14.6) and 49.5 (14). When a Bonferroni modified *t*-test was used to compare the mean values of HDL-c between the regimens, there were statistically-significant differences between dark chocolate × yerba mate (*p* = 0.04) and dark chocolate × placebo mate (*p* = 0.01), but no differences between dark chocolate × placebo chocolate (*p* = 0.09), yerba mate × placebo mate (*p* = 0.37), yerba mate × placebo chocolate (*p* = 0.20) and placebo mate × placebo chocolate (*p* = 0.70). When a paired Student *t*-test was used for comparisons between mean HDL-c at baseline and after receiving the different supplements, there was a statistically-significant difference between HDL-c at baseline and after supplementation with dark chocolate (*p* = 0.008), but no difference between HDL-c at baseline and after supplementation with placebo chocolate (*p* = 0.14). A *p*-value ≤ 0.05 was considered significant.

## 4. Discussion

### 4.1. Chocolate Consumption

The present study showed statistically-significant differences among consumption of dark chocolate, placebo-chocolate, yerba mate and placebo-mate (*p* = 0.047). There was no statistically-significant difference between mean values of HDL-c after supplementation with dark chocolate and placebo-chocolate (*p* = 0.09) using the Bonferroni modified *t*-test, but a statistically-significant difference between the two regimens when using the paired Student *t*-test (*p* = 0.043). Compared to baseline values, the consumption of 65 g of dark chocolate increased the mean concentration of HDL-c (*p* = 0.008), but consumption of placebo-chocolate did not increase the mean concentration of HDL-c (*p* = 0.14). No changes were observed in hs-CRP, fibrinogen, TBARS or white blood cells.

HDL particles are known to exert anti-inflammatory effects in the organism, while LDL-c, when oxidized, causes an increase in oxidative stress and the release of inflammatory markers [5,6]. This manifestation of dyslipidemia is common in individuals with HIV/AIDS, and these findings need to be monitored regularly, since it is well established that lipid profile changes occur during the atherosclerotic process [31,35,36]. One of the mechanisms whereby HDL-c improves the inflammatory and oxidative profile is by inhibiting the oxidation of LDL-c through enzymes associated with its apoproteins, such as paraxonase-1, which can prevent or even reverse the oxidative process. These enzymes reduce the expression of adhesion molecules on the surface of endothelial cell membranes, which are responsible for the adhesion of leukocytes to the vascular endothelium during the early cascade of events that lead to processes of inflammation [37].

Using similar food supplements and time of administration, Mursu *et al*. [18] administered 75 g dark chocolate or dark chocolate enriched with polyphenols to healthy subjects for three weeks and observed an increase in HDL-c concentrations (11% and 14% for dark chocolate and enriched dark chocolate, respectively). In a clinical trial involving eutrophic Japanese men who consumed 26 g cocoa powder/day for 12 weeks, Baba *et al.* [38] found a significant increase in plasma HDL-c levels (23.4%).

### 4.2. Yerba Mate Consumption

In the present study, the administration of yerba mate did not cause alterations in the oxidative or inflammatory parameters analyzed. In an *in vivo* study including 32 rats divided into four groups (control, control-mate, hypercholesterolemic and hypercholesterolemic-mate), Mosimann *et al.* [39] also observed no difference in HDL-c, TBARS or antioxidant enzyme activity, although a larger quantity of yerba mate and a longer time of administration were used (two months of treatment with 400 mL tea extract/day). However, there was a reduction of the atherosclerotic area in the hypercholesterolemic-mate group. On the other hand, Matsumoto *et al.* [40], studying 15 healthy women instructed to consume 5 g instantaneous yerba mate diluted in 500 mL water once a day for seven days, observed improvement in the oxidative profile indicated by the decrease in TBARS values after acute (one hour of ingestion) and chronic administration (seven days of ingestion) of yerba mate. Apparently, the time of administration and/or concentration can interfere with the results, since Matsumoto *et al.* [40] used a shorter intervention period and a higher concentration of yerba mate compared to the present study. Another important factor is the study of populations with different characteristics. For example, the metabolism of individuals with HIV/AIDS may interact differently with polyphenols.

Studies on animals and humans investigating the consumption of yerba mate have used higher quantities of this beverage (400 mL to 1 L/day) and longer periods of administration ranging from 7 to 90 days [39,40,41]. In this clinical trial, a lower amount of the beverage (approximately 200 mL) was offered because of possible difficulties in adhering to the intervention, considering that the subjects were asked to consume four different supplements over a period of 15 days each, intercalated with 15 days of washout.

Based on literature data, it can be concluded that the consumption of yerba mate seems to have beneficial effects on some inflammatory and oxidative parameters in healthy subjects and patients with cardiovascular diseases. However, no effect of the administration of yerba mate was observed in the present study, a finding that could be explained by the time of administration,the amount of mate consumed and differences between populations, since this is the first study involving HIV-positive individuals.

### 4.3. hs-CRP

The mean hs-CRP values observed in the present study indicate that most participants were at medium and high cardiovascular risk [25]. According to Lau *et al.* [42], hs-CRP values of 2.3 mg/L or higher reduce by about half the time between HIV infection and the development of AIDS. All participants exhibited mean hs-CRP values higher than 2.3 mg/L throughout the study, demonstrating the even stable HIV-positive individuals with undetectable viral load and good adherence to ART have chronic inflammation and are at risk of developing AIDS.

### 4.4. Fibrinogen

Mean fibrinogen levels were within the recommended limits. According to Bo *et al.* [43], adipose tissue and LDL-c concentrations are associated with plasma fibrinogen levels, a fact that explains in part the results obtained, since most participants had BMI and LDL-c values within the normal range [31]. The mechanism underlying the association between adipose tissue and concentrations of acute-phase reactants is probably related to the secretion of IL-6 by adipose tissue, an interleukin involved in the regulation of the synthesis of fibrinogen and CRP. Another factor that may have influenced the results is that most participants in the study were Caucasians. Omoregie *et al.* [44] reported mean fibrinogen levels above those found in this study for black individuals with HIV (513 ± 1.9 mg/dL).

### 4.5. TBARS

Quantification of TBARS is used as a measure of lipid peroxidation [45]. In a prospective study involving subjects without HIV, Tanaka *et al.* [46] found a 3.2-times higher relative risk of myocardial infarction or cerebrovascular accident for subjects in the higher tertiles of TBARS (>5.3 μmol/L) compared to those in the lower tertiles (<4.2 μmol/L). The participants in the present intervention had high plasma TBARS levels, with a mean of 14.5 ± 4.9 μmol/L throughout the study. In a study conducted in São Paulo, healthy subjects aged 40 to 49 years had a mean TBARS level of 2.3 μmol/L [47]. The results obtained here are higher even when compared to studies involving HIV-positive individuals undergoing ART [48]. This fact may be explained by the long time of exposure of the subjects to the virus and to ART.

### 4.6. White Blood Cell Count

The mean leukocyte count of the participants of this study remained within the normal limits throughout the study. Polymorphonuclear (PMN) cells account for 50% to 60% of all white blood cells and act directly at the site of inflammation. Neutrophils are the most abundant of these cells. Continuous hyperactivation of PMN cells has been observed in HIV-positive patients undergoing ART, which may be related to the imbalanced necrosis/apoptosis mechanism observed in this population. This imbalance compromises phagocytosis by PMN cells, which is associated with chronic inflammation in HIV-positive individuals [49].

There is a divergence in the literature regarding the effect of phenolic compounds on PMN cells. Kenny *et al.* [50] demonstrated in an *in vitro* study that flavonoids and procyanidins isolated from cocoa can modulate the oxidative and inflammatory response mediated by PMN cells. The mechanism proposed is the inhibition of ROS by lipopolysaccharides through the modulation of adhesion molecules that contribute to the activation of PMN cells. In a cross-over study with a longer intervention period, but similar quantity of chocolate (70 g bitter chocolate for four weeks) as that employed in the present study, Esser *et al.* [51] observed that chocolate consumption reduced total leukocyte count, as well as the concentrations of the adhesion molecules sICAM1 and sICAM3 and markers of leukocyte adhesion. The consumption of chocolate also reduced the expression of CD66b and CD11c surface proteins on neutrophils. These proteins are involved in the recruitment and adhesion of leukocytes to the endothelium during the first steps of atheromatous plaque formation, reducing atherogenic symptoms and inflammatory stimulation. Reduced activation of monocytes and neutrophils was also observed in an *in vivo* study conducted by Heptinstall *et al.* [52] using a cocoa beverage rich in phenolic compounds. However, that study suggested that cocoa constituents other than phenols might have contributed to the result observed. In contrast, Davison *et al.* [53] found no change in the magnitude of the leukocyte and neutrophil response in subjects consuming 100 g dark chocolate two hours before physical activity.

Changes in the concentration of leukocytes and neutrophils are also influenced by external factors, such as stress, tobacco and alcohol consumption and intense physical activity, and by internal factors, such as the production of corticosteroids and adrenalin.

### 4.7. Limitations of the Study

Possible limitations of the study were: (1) loss of part of the sample;and (2) additional chocolate or yerba mate intake. According to Fewtrell *et al.* [54], evaluation of the impact of the loss to follow-up by comparing the characteristics of the lost population and the population included in the study is an approach to avoid selective survival bias. Analysis of the lost sample showed that it generally had the same characteristics as the population that remained in the study, except for age,which differed between groups. Although age is a factor that can influence inflammatory responses, a comparison of mean hs-CRP, fibrinogen and HDL-c levels showed a similar inflammatory profile in the two groups. The fact that the subjects were their own controls minimizes bias associated with the loss to follow-up. Another possible limitation of the study is related to additional chocolate or yerba mate intake. This issue can be a problem in clinical trials that deal with food supplementation. However, in this study, the participants were advised to eat chocolate in replacement of a dessert, given the high caloric intake of the product, and to drink yerba mate in replacement of a refreshment.

### 4.8. Forces of the Study

The design of the study was appropriate, and the order of receiving the supplements was randomized. Furthermore, the trial was not biased from carryover effects, considering that there were washout periods between intakes of the supplements. Therefore, the study complied with the requirements of cross-over trials [55].

## 5. Conclusions

This is the first clinical study in the international literature that evaluated the effect of chocolate and yerba mate consumption on the inflammatory and oxidative profile of individuals with HIV/AIDS undergoing ART. The results showed that the consumption of 65 g dark chocolate (average of 2148 mg total phenols) was sufficient to increase HDL-c concentrations.

Supplementation with 3 g soluble yerba mate (average of 107 mg total phenols and 84.24 mg chlorogenic acid) for 15 days did not change inflammatory or oxidative markers and the parameters in these subjects. The results may be due to metabolic changes caused by the infection itself, as well as to the use of ART, the period of intervention, the amount of supplement offered and the concentration of polyphenols in the supplements, which may not have been sufficient to produce important changes in the outcome variables. Furthermore, there is no consensus in the literature regarding the bioavailability of polyphenols present in supplements and other factors that influence polyphenols activity, such as an altered intestinal microbiota in individuals with HIV/AIDS.

## Figures and Tables

**Table 1:** Comparison of the characteristics of individuals with HIV/AIDS undergoing antiretroviral therapy who completed the study (*n* = 92) and those who did not (*n* = 62).

Variable	Completed the Study *N* (%)Mean (SD)		Did Not Complete the Study *N* (%)Mean (SD)		*p*-Value
Gender					0.63
Male	58 (63.0)		36 (58.1)		
Female	34 (37.0)		26 (41.9)		
Age (years)	92 (100)	45.0 (7.1)	62 (100)	41.0 (8.1)	0.00
Race/ethnicity					
White	43 (46.7)		39 (62.9)		0.12
Black	16 (17.4)		6 (9.7)		
Mixed race	31 (33.7)		17 (27.4)		
Schooling (years)	92 (100)	5.0 (1.6)	62 (100)	5.0 (1.7)	0.92
Per capita income (US$)	92 (100)	465.6 (477.4)	62 (100)	378.1 (321.0)	0.50
Smoking					0.14
Yes	16 (17.4)		19 (30.7)		
No	61 (66.3)		36 (58.1)		
Ex	15 (16.3)		7 (11.3)		
Alcohol ingestion					0.56
Yes	46 (50)		28 (45.2)		
No	46 (50)		34 (54.8)		
BMI (kg/m^2^)	92 (100)	23.4 (3.9)	62 (100)	23.4 (3.4)	0.96
Body fat (%)	92 (100)	19.8 (9.1)	62 (100)	21.2 (9.5)	0.33
CD4+ T lymphocytes (cells/mm^3^)	92 (100)	596.1 (263.4)	62 (100)	547.1 (281.5)	
Time since HIV diagnosis (years)	92 (100)	13.32 (5.1)	62 (100)	11.9 (5.2)	0.07
Time since onset of ART (years)	92 (100)	10.7 (5.2)	62 (100)	9.4 (4.9)	0.08
Adherence to ART					0.16
Yes	88 (95.7)		56 (90.3)		
No	4 (4.4)		6 (9.7)		
hs-CRP (mg/dL)		3.0 (8.3)		3.1 (8.5)	0.74
<1.0 (low risk)	31 (33.7)		19 (30.6)		
1.0│-│ 3.0 (average risk)	40 (43.5)		26 (41.9)		
>3.0 (high risk)	21 (22.8)		17 (27.4)		
Fibrinogen (mg/dL)		350.6 (121.6)		346.6 (116.6)	0.18
150│-│ 400 (appropriate)	66 (71.7)		44 (70.9)		
>400 (high)	26 (28.3)		18 (29.0)		
Total cholesterol	92 (100)	193.2 (35.2)	62 (100)	188.7 (30.8)	0.09
HDL-c (mg/dL)		49.8 (14.8)		44.8 (14.5)	0.06
Men					
<40 (low)	22 (37.9)		15 (41.6)		
≥40 (appropriate)	36 (62.1)		21 (58.3)		
Women					
<50 (low)	13 (38.2)		9 (34.6)		
≥50 (appropriate)	21 (61.8)		17 (65.3)		
LDL-c (mg/dL)	92 (100)	114.4 (30.7)	53 (85.5)	117.9 (39.5)	0.65

BMI, body mass index; HIV, human immunodeficiency virus; ART, antiretroviral therapy; hs-CRP, high-sensitivity C-reactive protein; HDL-c, high-density lipoprotein cholesterol; LDL-c, low-density lipoprotein cholesterol. Alcohol ingestion: once or more per week.
